# Pomegranate Juice Enhances Healthy Lifespan in *Drosophila melanogaster*: An Exploratory Study

**DOI:** 10.3389/fpubh.2014.00245

**Published:** 2014-12-16

**Authors:** Subramani Paranthaman Balasubramani, Jayaram Mohan, Arunita Chatterjee, Esha Patnaik, Subrahmanya Kumar Kukkupuni, Upendra Nongthomba, Padmavathy Venkatasubramanian

**Affiliations:** ^1^Foundation for Revitalisation of Local Health Traditions (FRLHT), Bangalore, India; ^2^Manipal University, Manipal, India; ^3^Department of Molecular Reproduction, Development and Genetics, Indian Institute of Science, Bangalore, India

**Keywords:** pomegranate, anti-aging, rasayana, ayurveda, *Drosophila*

## Abstract

Exploring innovative ways to ensure healthy aging of populations is a pre-requisite to contain rising healthcare costs. Scientific research into the principles and practices of traditional medicines can provide new insights and simple solutions to lead a healthy life. Rasayana is a dedicated branch of Ayurveda (an Indian medicine) that deals with methods to increase vitality and delay aging through the use of diet, herbal supplements, and other lifestyle practices. The life-span and health-span enhancing actions of the fruits of pomegranate (*Punica granatum* L.), a well-known Rasayana, were tested on *Drosophila melanogaster* (fruitfly) model. Supplementation of standard corn meal with 10% (v/v) pomegranate juice (PJ) extended the life-span of male and female flies by 18 and 8%, respectively. When male and female flies were mixed and reared together, there was 19% increase in the longevity of PJ fed flies, as assessed by MSD, the median survival day (24.8). MSD for control and resveratrol (RV) groups was at 20.8 and 23.1 days, respectively. A two-fold enhancement in fecundity, improved resistance to oxidative stress (H_2_O_2_ and paraquat induced) and to *Candida albicans* infection were observed in PJ fed flies. Further, the flies in the PJ fed group were physically active over an extended period of time, as assessed by the climbing assay. PJ thus outperformed both control and RV groups in the life-span and health-span parameters tested. This study provides the scope to explore the potential of PJ as a nutraceutical to improve health span and lifespan in human beings.

## Introduction

Global statistics predict 223% increase in the number of elderly (aged >60 years) accounting for about 1.2 billion between 1970 and 2025 (1). Ways to ensure healthy aging while increasing life-span are heatedly debated and researched in public health circles (2). This is because long-term institutional care of the elderly for chronic age-related conditions such as arthritis and dementia has led to steep increases in healthcare costs, yet reduced the quality of life for the patients (3, 4). Countries have started recognizing that promoting self-reliant wellness strategies among communities would not only save the exchequer from substantial financial burden due to illness care but also have a population that is healthy and productive (5). Increasing evidence through longitudinal studies show that simply adopting appropriate diet and lifestyle can even reverse chronic conditions like coronary atherosclerosis (6).

Complementary and alternative medicines like ayurveda, yoga, and traditional Chinese medicine prescribe several interventions for prevention of diseases and promotion of health that can be scientifically explored for wellness. *Rasayana* is a special branch of Ayurveda, which deals with methods of rejuvenation such as dietary recipes and regimen, herbal and mineral supplements, and health-promoting lifestyle that are said to enhance quality of life and delay aging (7). Traditional literature describes Rasayanas as methods of reversing naturally occurring senility and improving mental competence (8), increasing immunity against diseases and providing vitality and luster to the body (7). As per Ayurveda, Rasayanas act on the body by (i) improving the process of digestion and metabolism (*Agni*), leading to better bio-availability, (ii) increasing the micro-circulation of nutrients through the body channels (*Srotas*), and (iii) enhancing the nutritive value of the plasma that gets generated (*Rasadhatu*) (7, 9). Several studies have indicated Rasayanas to be anti-oxidants and immunomodulators (10).

Ayurveda identifies several fruits and herbs including the Indian Gooseberry (*Phyllanthus emblica*), Pomegranate (*Punica granatum*), Drumsticks (*Moringa oleifera*), Long pepper (*Piper longum*), etc., as Rasayanas (7, 8, 10). Understanding the scientific basis behind the concept of Rasayana and validation of the life promoting ability of commonly available Rasayana fruits and herbs can help establish holistic and simple ways to wellness through diet.

The potential therapeutic properties of pomegranate, a commonly available fruit, have been studied for wide-ranging applications including cardiovascular disease, diabetes, dental conditions, erectile dysfunction, protection from ultraviolet (UV) radiation, infant brain ischemia, prevention of cancer, Alzheimer’s disease, male infertility, arthritis, and obesity (11–13). Pomegranate is considered as a symbol of life, health, longevity, fecundity, intellect, immortality, and spirituality by Unani and Traditional Chinese Medical systems as well (14). Ayurvedic texts describe pomegranate as a “wholesome” (*Pathya*) fruit with several properties including tissue generation and development and strength promotion (15). It is particularly indicated for use in cardiac and anemic conditions (16).

Aging is a complex physiological phenomenon, particularly because it manifests itself over a wide range of biological systems, tissues, and functions. Aging pathways are highly conserved across species including *Saccharomyces cerevisiae*, *Caenorhabditis elegans*, *D. melanogaster*, and rodents (17, 18). *Drosophila* (commonly known as fruit fly) has many advantages for studying the biology of aging. For example, flies show physiological, genetic, and anatomical similarities with human (17). Owing to short-life-span and ease of handling, the fruit flies are particularly ideal for conducting prevention and wellness studies, which would otherwise take several years in human beings (17). Fruits like cranberry (19), blueberry (20), orange, lemon (21), apple polyphenols (22), and cocoa (23) have been shown to enhance lifespan in *Drosophila* model. A few Ayurvedic formulations have also been tested for their bioactive potential in the *Drosophila* model (24, 25).

Pomegranate, a member of the Punicaceae family is native to the Himalayas. However, it is also under commercial cultivation in other parts of India and the world (12). It contains polyphenols such as ellagitannins and anthocyanins, as well as phenolic acids, fatty acids, alkaloids, and a variety of volatile compounds (26). Ellagitannins are among the most prevalent compounds present in pomegranate and may be responsible for various bioactivities (27). The juice contains ellagic acid, gallic acid, anthocynidins, flavan-3-ols, straight chain fatty acids, citric acid, and malic acid besides glucose, fructose, and sucrose as the major sugars (26). Pomegranate has been shown to have anti-oxidant, anti-inflammatory, lipid regulation, gastroprotective, anti-diarrhea, anti-cancer, anti-viral, anti-angiogenesis, anti-diabetic, and anti-atherosclerotic activities (26, 27).

The Rasayana actions of pomegranate, namely, enhancement of life-span, reproductive ability, stress tolerance, resistance to infection, and stamina were compared between control, resveratrol (RV), and pomegranate juice (PJ) supplemented *D. melanogaster*. Our hypothesis was that pomegranate supplementation would significantly improve the performance of the flies on all counts, as compared to the control.

## Materials and Methods

### *Drosophila* strain, media, and culture conditions

*Drosophila melanogastor* wild type, *Canton-S* strain, was used for this study. The basal media for culturing the flies consisted of corn flour (60 g), sugar (20 g), d-glucose (20 g), dry yeast (15 g), and agar (8 g) per liter. After cooking, media was allowed to cool to room temperature and then propionic acid (4 ml), benzoic acid (0.7 g in 5 ml of ethanol), orthophosphoric acid (0.6 ml) were added and mixed thoroughly. Media cooked as slurry was poured into the bottles or vials and was allowed to solidify before transfer of adult flies or pupae. Stock flies were maintained in bottles while culture vials were used for maintenance of smaller populations or experimental groups. Flies were allowed to feed *ad libitum* and grown at 25°C with 60% humidity in 12 h day–night cycle.

### Preparation of pomegranate juice and resveratrol stock

Pomegranate juice was prepared by hand crushing the arils of fresh fruits through sterile muslin cloth. The juice was passed through a filter (0.2 μm; Millex, Millipore, Germany) and stored as aliquots of different volume (5, 15, and 50 ml) in screw capped tubes at −80°C until use. Freezing did not significantly alter the high-performance liquid chromatography (HPLC) profile (data not shown). RV (Sigma-Aldrich, St. Louis, MO, USA) stock (100 mM) was prepared by dissolving 22.8 mg in 1 ml of ethanol. Both PJ and RV were added to the cooked and warm basal media before dispensing into vials.

### Lifespan extension experiment

Age matched (2–3 days) flies were generated by removing all adult flies in the bottles. Once a synchronous culture was generated, sex separated flies were transferred into vials containing basal medium supplemented with 0.1, 1, 5, 10, and 15% (v/v) of PJ. Flies were transferred to fresh media every third day. Dead flies were counted and removed daily until the death of the last fly in each vial. PJ at 15% v/v could not be used since at that concentration the medium would not set.

### Longevity and fecundity analysis

Synchronous cultures of 2–3-day-old files were obtained as described earlier and transferred into vials containing basal medium supplemented with 10% of PJ (v/v) or 200 μM RV and control. Each group including the control had 10 vials each with 14–20 flies per vial with equal sex ratio. Flies were transferred to fresh media every third day. Dead flies were counted and removed daily throughout the experiment. New flies emerging in the emptied vials were counted daily for 10 days from the day of the emergence of the first fly in each experimental group.

### Gustatory assay

The method described by Lee et al. (28) was adopted for gustatory assay. Adult flies were reared for 20 or 40 days in media supplemented with 10% PJ or 200 μM RV. A basal media control was also maintained. To perform a feeding assay, after starving the flies for 2 h, 30 male or female flies from each experimental group were transferred into the vials containing the specific diets with bromophenol blue dye (0.05% wt/vol) (Sigma-Aldrich, St. Louis, MO, USA). After 10 min of feeding, the fed flies were etherized, washed with phosphate-buffered saline (PBS), and homogenized in 1 ml of distilled water. The absorbance of 100 times diluted supernatant was measured at 595 nm using a spectrophotometer (Bio-Rad, CA, USA).

### Resistance against hydrogen peroxide and paraquat induced stress

The method described by Peng et al. (22) was used for studying the fly’s ability to resist free radical stress induced by H_2_O_2_ (Fisher Scientific, Mumbai, India) and paraquat (Sigma-Aldrich, St. Louis, MO, USA). H_2_O_2_ generates hydroxyl radical (OH*) while paraquat produces superoxide (O*) radicals. Twenty days old male and female flies from control, RV (200 μM), and PJ (10%) fed were starved for 2 h and transferred to separate vials (*n* = 50; 10 flies/vial) containing 5% H_2_O_2_ or 20 mM paraquat prepared with 5% sucrose on saturated tissue paper mat. Dead flies were counted every 4 h until the death of the last fly.

### Resistance against infection

The method described by Apidianakis and Rahme (29) was followed with slight modifications. Antigen was prepared by suspending an overnight culture of *C. albicans* SC5314 in sterile distilled water (2 × 10^8^ cells/ml – as counted using a hemocytometer; data not shown). Flies (20 days old) were anesthetized with CO_2_. Infection was induced with a sterile tungsten needle (0.01 mm diameter) dipped in the *C. albicans* suspension by gently pricking in the thoracic region. Flies were returned to their respective food vials and were incubated at 25°C with 12 h day–night cycle until 50% of flies died in any of the groups. Control flies were pricked with needle dipped in sterile distilled water.

### Climbing assay

On day 20 and 40, locomotor function of fruit flies was assessed using the climbing or negative geo-taxis assay as reported by Bahadorani and Halliker (23) with slight modifications. In brief, 20 flies/trial were placed in the bottom of a measuring cylinder and given 20 s to climb up. At the end of each trial, the number of flies that climbed up to a vertical distance of >8 cm was recorded. Each trial was repeated three times.

### Statistical analysis

The day when 50% of the total flies in each of the experimental group survived was calculated as the median survival day. Log rank test^1^ was used for survival analysis while Wilcoxon rank sum test^2^ was used for comparing the life-span extension (30). Student’s *t*-test was used for comparison of means. ANOVA was employed while comparing more than two groups. *p*-Value <0.05 was considered as significant.

## Results

### PJ supplementation extends lifespan in *Drosophila*

**Figure 1:**
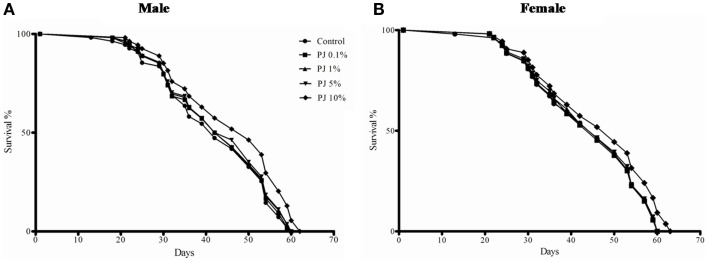
**Life-span extension by different concentrations of pomegranate juice (PJ) in *Drosophila* (A) male flies and (B) female flies**. Ten percent PJ substitution to the media significantly increased (*p* < 0.00001) the survival of the flies and extended their life-span by 2 days. In both the sexes 0.1, 1, or 5% PJ did not show any enhancement in the survival.

To determine the lifespan extending potential in *Drosophila*, we supplemented the basal media with 0.1, 1, 5, 10, and 15% of PJ. Substitution of the media with 10% PJ significantly enhanced the median lifespan by 18.51 and 8% in male and female flies, respectively (Figure 1; Table 1). Last fly survival day was also enhanced by 2 days in the 10% PJ group. While the lower concentration tested (0.1, 1, and 5%) did not produce any significant changes in the life-span, 15% PJ substitution created a practical difficulty in solidification of the medium; therefore, this concentration was not considered. Thus, 10% PJ was used in all further experiments.

**Table 1:** **Statistical analysis of the survival curves using Log rank tests and Wilcoxon rank sum tests**.

Experimental group	Number of flies tested (*n*)	Median survival day/hour	% Change	Chi square value @ 1 degree of freedom	Log rank (survival)	Wilcoxon (maximum life-span)
**Lifespan extension**
Male
Control	110	40.5				
PJ 0.1	108	42.0	3.7	0.7	*p* = 0.396	*p* ≤ 0.6421
PJ 1	112	42.0	3.7	1.6	*p* = 0.207	*p* ≤ 0.5322
PJ 5	108	42.0	3.7	3.9	*p* = 0.047	*p* ≤ 0.6208
PJ 10	108	48.0	18.51	46.2	*p* < 0.00001*	*p* ≤ 0.2545
Female
Control	104	43.5				
PJ 0.1	106	43	−1.14	0.1	*p* = 0.699	*p* ≤ 0.6861
PJ 1	112	43.5	0	0.1	*p* = 0.720	*p* ≤ 0.5084
PJ 5	112	44	1.14	0.1	*p* = 0.758	*p* ≤ 0.5392
PJ 10	108	47	8.0	19	*p* < 0.00001*	*p* ≤ 0.3439
**Longevity and fecundity**
Control	247	20.8				
RV	167	23.1	11.05	16.2	*p* < 0.00001*	*p* ≤0.6016
PJ	164	24.8	19.23	48.7	*p* < 0.00001*	*p* ≤0.3506
**H_2_O_2_ stress survival**
Male
Control	50	18.0				
RV	50	24.5	36.1	29.3	*p* < 0.00001*	*p* ≤ 0.4705
PJ	50	28.0	55.5	1.6	*p* < 0.00001*	*p* ≤ 0.2602
Female
Control	50	19.0				
RV	50	26.5	39.4	22.5	*p* < 0.00001*	*p* ≤ 0.4237
PJ	50	29.5	55.2	49.4	*p* < 0.00001*	*p* ≤ 0.1824
**Paraquat stress survival**
Male
Control	50	15.5				
RV	50	17.0	9.6	7.4	*p* = 0.0066*	*p* ≤ 0.6295
PJ	50	24.0	54.8	19.2	*p* < 0.00001*	*p* ≤ 0.4907
Female
Control	50	18.5				
RV	50	24.5	32.4	8.7	*p* = 0.0032*	*p* ≤ 0.4553
PJ	50	27.0	45.9	30.3	*p* < 0.00001*	*p* ≤ 0.2856

	***n***	**Median survival time (MST) in hours**	**% MST increase**	**% of surviving flies**	**ANOVA**	***t*-test**

**Resistance against infection**
Male
Control	50	4.88		41	*p* = 0.0013*	
RV	50	>6	>23	55		*p* ≤ 0.05*
PJ	50	>6	>23	64		*p* ≤ 0.05*
Female
Control	50	3.82		38	*p* = 0.0012*	
RV	50	>5	>31	59		*p* ≤ 0.05*
PJ	50	>5	>31	78		*p* ≤ 0.05*

*Significant.

### PJ supplementation controls the “trade-off” between fecundity and longevity

As 10% PJ substitution to the media significantly enhanced life-span for both male and female flies separately, we investigated the effect of this concentration in the mixed group of male and female. RV at 200 μM dose was used as a positive control, based on the reports of Bass et al. (31).

**Figure 2:**
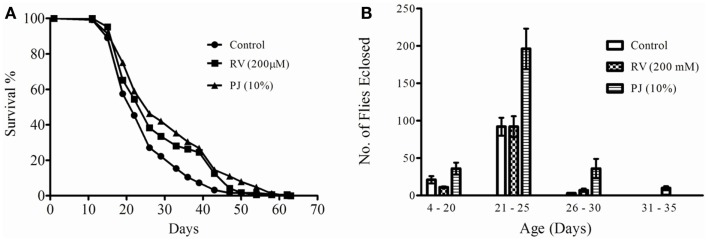
**(A)**. Effect of pomegranate juice supplementation on *Drosophila* longevity. Male and female mixed culture flies were fed with 10% PJ (*n* = 164) or 200 μM RV (*n* = 167). A basal medium control (*n* = 247) was also maintained. Log rank test indicated significant (*p* < 0.00001) increase in the fly survival by both PJ and RV (Table 1). But PJ increased the median survival by 19.23% while it was 11.05% in the RV group. **(B)** Effect of 10% pomegranate juice supplementation on *Drosophila* fecundity at different age. The bars represent the total number of flies enclosed from each group at different age with SD. PJ group showed significant increase in the number of off-springs produced (*p < 0.0001). The graph also indicates an extended reproductively viable phase in the PJ fed flies. No significant difference in fecundity was observed with RV and control groups.

Pomegranate juice enhanced the median survival of a mixed population of flies from 20.8 (as seen in control) to 24.8 days (PJ), i.e., PJ enhanced median life-span by 19.23% more than the control (*p* < 0.00001) (Figure 2A; Table 1). RV group on the other hand enhanced the median survival to 23.1 days, which was only 11% over the control group (Figure 2A; Table 1). The maximum life-span observed in the control and PJ groups was 61 days while that in the RV group, the last fly survived till 62nd day. Reproductive potential of an organism also indicates its physiological fitness. So, we monitored the number of flies emerging from each vial of PJ, RV, and control group. The data shown in Figure 2B indicate that PJ group produced significantly (*p* < 0.0001) more number of flies throughout the reproductive phase. Also, the new fly emergence data indicated that PJ extended the reproductive viability phase (Figure 2B).

### *Drosophila* has the same feeding preference to PJ and RV supplementation

**Figure 3:**
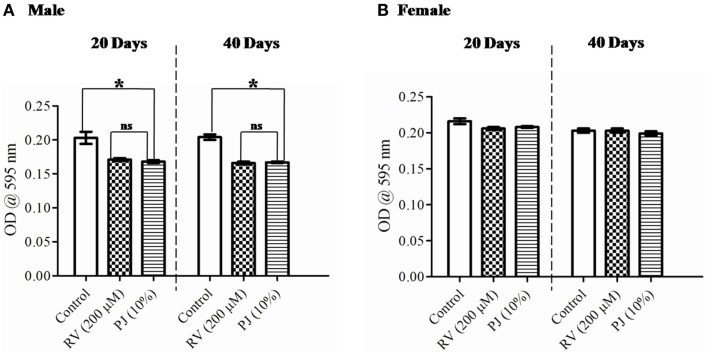
**Gustatory assay with PJ (10%) or RV (200 μM) supplementation in 20 and 40 days old *Drosophila* flies**. **(A)** Male flies in the PJ and RV group were found to have reduced feed intake when compared to control (*p < 0.05). However, there was no difference in food intake by PJ and RV groups. **(B)** Female flies did not show any significant difference in the food intake on both the days tested (ns, not significant).

Feeding behavior and nutritional constituents of a culture medium are important factors in the life-span determination of *D. melanogaster* (30). Bass et al. (31) has reported that RV enhances lifespan by dietary restriction in *Drosophila* and *C. elegans*. To ensure that the observed changes in the lifespan were due to PJ or RV supplementation and not because of starvation or dietary restriction, gustatory assay was performed, quantifying the food intake on 20- and 40-day-old flies using bromophenol blue dye. While there was no significant difference observed between PJ and RV fed groups, the feed intake was lower in both these groups as compared to that in the control (*p* < 0.05), particularly in the male flies (Figure 3A). This difference was not significant in the female flies. The feed intake of 40-day-old female flies was similar in control, PJ, and RV groups (Figure 3B). Also, there was a significant difference (*p* < 0.05) in food intake between the male and female flies in both PJ and RV groups (Figure 3).

### PJ enhances tolerance to free radical induced stress

**Figure 4:**
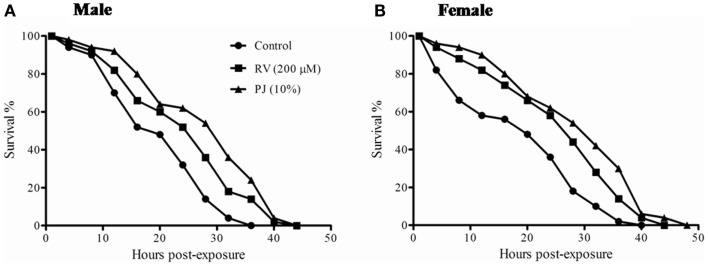
Survival against hydroxyl radical (OH*) produced by hydrogen peroxide exposure in 20 days old *Drosophila*. Both male **(A)** and female **(B)** flies fed with PJ (10%) showed approximately 50% increase in median survival (*p* < 0.00001) (Table 1). RV (200 μM) fed flies showed only about 30–35% increase in median survival.

**Figure 5:**
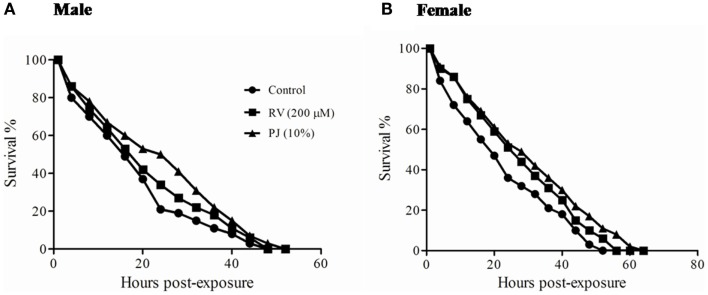
Survival against superoxide radical (O*) produced by paraquat exposure in 20 days old *Drosophila*. **(A)** PJ (10%) group showed 54% increase in median survival (*p* < 0.00001) while RV (200 μM) could increase only by 9.6% (Table 1). **(B)** Female flies fed with PJ showed 45.9% increase in median survival (*p* < 0.00001) while RV was able to increase the median survival by 32.4%.

Ayurveda claims that Rasayanas improve the stress resistance capacity of organisms. Both H_2_O_2_ and paraquat produce free radicals and are lethal to flies. A significant (*p* < 0.00001) increase in the post H_2_O_2_ and paraquat exposure survival time was observed in both PJ and RV supplemented flies when compared to the control (Figures 4 and 5; Table 1). Analysis of median survival indicated an average 50% increase over the control group in both male and female flies in PJ. Though, RV was also found to protect against the free radical induced stress, its level of protection showed only about 30–35% increase in survival (Figures 4 and 5).

### PJ protects *Drosophila* from *Candida albicans* infection

**Figure 6:**
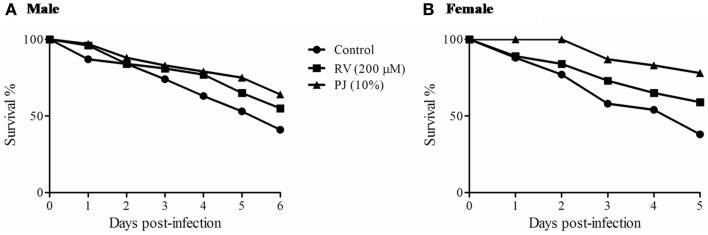
**Pomegranate protects *Drosophila* from *Candida albicans* infection**. Twenty days old male **(A)** and female **(B)** flies fed with PJ, RV, or control diet were infected with *Candida albicans*. In both the sexes, flies fed with PJ showed a significantly (*p* ≤ 0.05) better survival response than the control and RV fed.

*Drosophila melanogaster* has been used as a model to study host–fungal interaction and pathogenicity (32). Glittenberg et al. (33) established *Drosophila* as an alternative model for investigating the pathogenicity of *C. albicans*. Literature indicates that Rasayanas have immunomodulatory (*Vyadhikshamatwa*) potential (10). As proof of principle, we evaluated if PJ substitution offered protection to the flies from death because of *C. albicans* infection. The survival curve indicated a significant (*p* ≤ 0.05) protective effect of PJ until 144 h post-infection. Even though, RV too protected the flies (>50%) from *C. albicans* infection, the magnitude of protection by PJ (>70%) was significantly higher. The control flies had only 40% survival (Figure 6). While the magnitude of protection differs with male and female flies, the protection trend was similar.

### PJ promotes sustained physical performance in *D. melanogaster*

**Figure 7:**
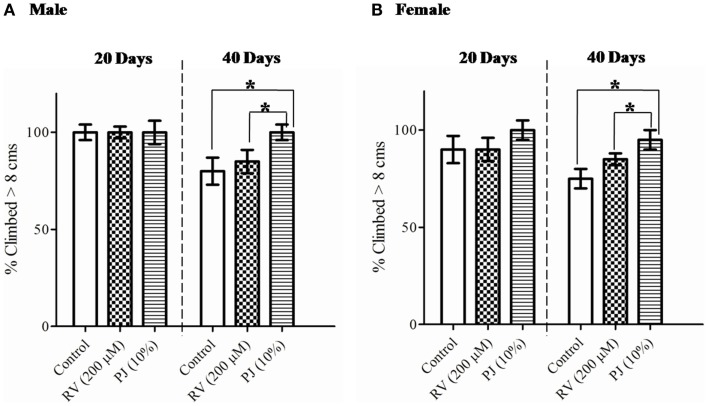
**Sustained climbing performance of *Drosophila* flies fed with PJ**. **(A)** Twenty days old male flies did not show any difference in their climbing performance irrespective of feed, but 40 days old PJ fed flies were able to retain their physical performance (*p* < 0.0001) then the RV and control group. **(B)** Female flies at both 20 and 40 days showed significantly higher (*p* < 0.0001) climbing performance then the RV and control group. PJ group was able to resist the age-related physical activity decline observed in the RV and control groups.

The success of healthy living lies in retention of normal physical activity. Rasayanas like pomegranate are said to improve energy, strength, and stamina (*Balya*) of organisms. The physical performance of the flies was measured using the climbing or negative geo-taxis assay. This assay was performed with 20 and 40 days old male and female flies fed on media supplemented with PJ or RV. Even though, significant difference among the groups was not observed in the percentage of flies climbing on 20th day, the PJ group retained the physical activity even on day 40 (aged flies), while the other groups showed severe decline (*p* < 0.05) (Figure 7). Thus, PJ was found to sustain good physical activity in terms of negative geo-taxis climbing activity during aging.

## Discussion

Developing scientifically validated, culturally acceptable, appropriate guidelines for healthy eating is recognized as one of the thrust areas for reducing age-related functional decline (1). An Australian longitudinal analysis to guide health promotion concludes that health, life style, and gender influences healthy aging (34). In Ayurveda, Rasayanas are said to improve immunity, impart vitality, are good aphrodisiacs and are considered to delay the aging process (8, 10). Pomegranate has been mentioned in Ayurveda as a Rasayana with the above properties, which was the reason for selecting the fruit for our study. We have shown that PJ fed flies live and reproduce longer (Figure 2B), are better protected against stress and infection (Figures 4–6) and are active even when aged (Figure 7). According to the antagonistic pleiotrophy theory, higher levels of reproduction are negatively correlated with survival. This concept of “trade-off” between longevity and reproduction or “cost of reproduction” has been widely accepted and demonstrated in a number of experimental studies (17). However, our observation in the current experimental setup indicated that PJ is capable of simultaneously enhancing fertility as well as survival. Reduction in the fertility of individuals has been considered as one of the reasons for accelerated graying in population (1). Ayurvedic texts indicate Rasayanas as holistic interventions that reduce age-related senility while maintaining physical and biological performance of the organism (7). This study opens up the scope of scientifically exploring Rasayanas to identify methods and to counter the burning contemporary issue of healthy aging.

Studies have shown that elevated resistance to various environmental stresses, including oxidative stress is one of the phenotypes of long lived drosophila mutants (22). When the protecting ability of PJ and RV was assessed against H_2_O_2_ and paraquat induced oxidative stress, we observed that PJ could play a role in ameliorating the free radical induced damage, which is one of the reasons for aging. The anti-oxidant potential of pomegranate demonstrated by Faria and Calhau (12) support our findings. Cranberry extract, apple polyphenols, curcumin, are some of the fruits and herbs that have been reported to impart protection against H_2_O_2_ and paraquat induced free radicals in *Drosophila* (19, 22, 28). Stress not only affects older individuals but it is also predisposed to cause premature aging (35). Free radicals and oxidative stress are increasingly being accepted as a major player in several disease processes and accelerated aging (36). Flavonoids and polyphenols in fruits like cranberry and blueberry have been shown to protect cells from oxidative stress induced damage caused by pro-inflammatory mediators (37). The polyphenol content in pomegranate may be responsible for imparting resistance against free radical induced stress (26). While fruit powders of blueberry and cranberry impart health benefits to flies at 2–3% w/v, orange juice was found to be effective at higher concentrations (3–50% v/v) (19, 20). Purified apple polyphenols (10 mg/ml of fly diet) were found to significantly enhance the health and life-span of flies. The concentration of polyphenols was estimated to be 0.4/100 kg of apple pomace (22).

*Drosophila*, although devoid of an adaptive immune system, harbors an innate immune response with striking similarities to the mammalian defense mechanisms. The *Drosophila* innate immune response uses pattern recognition receptors to activate phagocytosis by plasmatocytes, proteolytic clotting cascades in the hemolymph and production of specific antimicrobial peptides (AMPs) in the fly fat body (32). The expression of specific anti-fungal peptides is controlled by a signaling pathway orchestrated by the toll-like receptors (TLR) similar to human beings (38). The exact mechanism by which PJ exerted protection to the flies is yet to be elucidated. Infectious diseases have been posing a major threat to the elderly population because of age-related decline in immune functions (39). Such conditions can be overcome by use of immune-stimulators. Several Rasayanas like pomegranate can be simple interventions to enhance immunity. *Drosophila* has been established as a model to also study age-related impairment in physical movement (40). Present study indicates that pomegranate feeding can sustain physical movement in aged flies. The extended reproductive phase (Figure 2B) and sustained physical activity (Figure 7) in the flies can be considered as direct indicators of healthy life-span.

The current study corroborates earlier reports that RV enhances lifespan in *Drosophila* (18, 31, 41). In this study, we have observed that RV increased median life-span of *Drosophila* by 11% (Figure 2A). RV substitution imparted comparatively lesser protective ability against H_2_O_2_, paraquat, and infection stress to the flies when compared to PJ fed group. RV did not significantly alter the fecundity of the flies as compared to the control. Estimation of the feed intake showed a slightly reduced feed intake quantity (Figure 3) in both PJ and RV supplemented groups when compared to the control. Earlier reports also indicate the possible dietary restriction by RV (31). In our experiments, this observation was more marked in the male flies than in the females. Ayurveda literature on pomegranate mentions that pomegranate provides satiation (*Trupti*), i.e., the organism is satisfied with lesser intake of food when supplemented with PJ (15, 16). While there was no significant difference in the feed intake between RV and PJ fed groups, the latter outperformed the former in all life-span and health-span parameters tested.

Free radical induced stress tolerance, improved infection survival, and climbing performance assays show that, pomegranate supplementation helps in reducing age-related functional decline in the flies. This study justifies the Ayurvedic claims that pomegranate is a Rasayana by being a multi-functional health promoter in fruit fly model. Given the exploratory nature of our study and the large number of comparisons made, the results are certainly very interesting; and it has opened up avenue for further research. Further research needs to be done to study the effectiveness of PJ supplementation in human beings at the community setting. Scientific exploration of other Rasayanas as quality-of-life enhancers is a potential area for research. Studying the mode of action of PJ, identification of bioactive components and molecular targets would be of value to understanding aging and anti-aging process.

*Drosophila* has been a strong candidate as a model in contemporary aging research. *D. melanogaster* has also been used as a model by researchers to study Rasayanas. An insect specific Rasayana was developed that was found to increase lifespan by 50% in *Drosophila* model (24). Dwivedi et al. (25) reported that Amalaki Rasayana, an herbal formulation and Rasa-Sindoor an organo-metallic derivative of mercury were capable of increasing life-span and fecundity in fruit flies.

Aging population and the associated physical, physiological, and neurological deficiencies are being recognized as the major challenges for healthcare in the twenty-first century. While people are living longer; they are aging prematurely (42). Scientific exploration of Rasayana can provide affordable ways to delay aging and maintain good health.

## Author Contributions

Padmavathy Venkatasubramanian conceptualized the transdisciplinary research methodology, obtained fund support, and edited the manuscript. Upendra Nongthomba designed *Drosophila* based experiments, analyzed results, and edited manuscript. Subramani Paranthaman Balasubramani performed the experiments, analyzed, interpreted results, prepared, and edited the manuscript. Jayaram Mohan maintained the *Drosophila* stocks and assisted Subramani Paranthaman Balasubramani in experimentation. Esha Patnaik and Chatterjee Arunita standardized the infection and stress experimentation protocol and analysis of results. Subrahmanya Kumar Kukkupuni provided the Ayurveda knowledge.

## Conflict of Interest Statement

The authors declare that the research was conducted in the absence of any commercial or financial relationships that could be construed as a potential conflict of interest.

## Footnotes

^1^http://bioinf.wehi.edu.au/software/russell/logrank/

^2^http://www.fon.hum.uva.nl/Service/Statistics/Wilcoxon_Test.html
